# Supplementary material for: Application of a Caries Treatment Difficulty Assessment System in Dental Caries Management
Source: Int J Environ Res Public Health. 2022 Oct 28;19(21):14069. doi: 10.3390/ijerph192114069 (PMC9656365; doi:10.3390/ijerph192114069)
Supplement: Supplementary file 1 [file ijerph-19-14069-s001.zip › ijerph-1891792-supplementary.pdf]

Table S1. Dental Caries Treatment Difficulty Assessment [16]

Difficulty classification	Disease Indicators	Referral accommodations
Grade I	1) Class I and V (according to G.V.Black);<br>2) Superficial caries and intermediate caries;<br>3) Direct restoration of posterior teeth: composite resin restoration and amalgam restoration. Minimally invasive techniques: ART, preventive resin restoration (PRR), glass ionomer transition repair, enamel molding, and micro-polishing;<br>4) A history of restoration, but caries not affecting the old restoration;<br>5) 3 fingers wide of mouth opening;<br>6) Free of pharyngeal reflex;<br>7) Normal salivary secretion;<br>8) Free of Dental phobia;<br>9) Low and medium risk population (caries risk assessment);	Grade A dentists (general practitioners)
Grade II	**Any factor of below:**<br>1) Class II, III, IV and VI Root caries (involvinglabial/buccal surface)<br>2) Deep caries;<br>3) Composite resin restoration of anterior teeth;<br>4) Caries involving the old restoration or the first fracture of the old restoration;<br>5) 2 fingers wide of mouth opening;<br>6) Pharyngeal reflex;<br>7) Massive salivary secretion;<br>8) Dental phobia;<br>9) High risk population (caries risk assessment);	Grade B dentists (cariology specialists)
Grade III	**At least 2 factors of Grade II or any factor of below:**<br>1) Cavity on the 1/3 gingival side of the distal surface of posterior teeth. Attrition Cusp defect Severely defected crown Root caries (involving more than 2 surfaces) Rampant Caries;<br>2) Deep caries of immature permanent teeth;<br>3) Cosmetic restoration of anterior teeth: non-invasive esthetic restoration, minimally invasive layered restoration, minimally invasive CAD/CAM ceramic veneer restoration Inlay restoration of posterior teeth: composite resin inlay, CAD/CAM ceramic inlay restoration;	Grade C dentists (clinical experts in cariology)

	4) Old restoration falling off 2 or more times;<br>5) Mouth opening less than 2 fingers wide;<br>6) Strong pharyngeal reflex;<br>7) Excessive salivary secretion;<br>8) Extremely high risk population (caries risk assessment);

Table S2. Caries Risk Assessment according to CAMBRA [37]

Caries risk classification	Influencing Factors
	**Low risk and moderate risk was depended on the balance of Risk factors and Protective factors below:**
Low risk	**Protective Factors**:<br>**1)** Lives/work/school fluoridated community<br>**2)** Fluoride toothpaste at least once daily<br>**3)** Fluoride toothpaste at least 2 times daily<br>**4)** Fluoride mouthrinse (0.05% NaF) daily<br>**5)** 5,000 ppm F fluoride toothpaste daily<br>**6)** Fluoride varnish in latst 6 months<br>**7)** Office F topical in last 6 months<br>**8)** Chlorhexidine prescribed/used one week each of last 6 months<br>**9)** Xylitol gum/lozenges 4x daily last 6 months<br>**10)** Calcium and phosphate paste during last 6 months<br>Adequate saliva flow (> 1 ml / min stimulated)
Medium risk	**Risk Factors** (Biological predisposing factors):<br>**1)** MS and LB both medium or high (by culture)<br>**2)** Visible heavy plaque on teeth<br>**3)** Frequent snack (> 3 times daily between meals)<br>**4)** Deep pits and fissure<br>**5)** Recreational drug use<br>**6)** Inadequate saliva flow by observation or measurement (If measured, note the flow rate below)<br>**7)** Saliva reducing factors (medications/radiation/systemic)<br>**8)** Exposed roots<br>**9)** Orthodontic appliances
High risk	**Including any of the following factors could be identified as high risk**:<br>**1)** Visible cavities or radiographic penetration of the dentin<br>**2)** Radiographic approximal enamel lesions (not in dentin)<br>**3)** White spots on smooth surfaces<br>**4)** Restorations last 3 years
Extrem risk	High risk + severe salivary gland hypofunction

Table S3. Occupational Classification

Classification	Content
The first category	People in charge of state organs, party-masses organizations, enterprises and institutions
The second category	Professional and technical personnel
Third category	Managerial or non-front-line personnel and staff in concerned areas
The fourth category	Business, service industry personnel
The fifth category	Workers in agriculture, forestry, animal husbandry, fishing and water conservancy
The sixth category	Production, transportation equipment operators and staff in concerned areas
The seventh category	Military personnel
The eighth category	Other practitioners with an unrevealed classification
